# Differential regulation of immunoglobulin class switch recombination by the tumor suppressor p53

**DOI:** 10.1038/s41598-026-49435-w

**Published:** 2026-04-25

**Authors:** Kawtar Hanefioui, Audrey Dauba, Charlotte Payrault, Fadila Guessous, Ahmed Amine Khamlichi

**Affiliations:** 1 Institut de Pharmacologie et de Biologie Structurale (IPBS), CNRS UMR5089, Université de Toulouse, 205 Route de Narbonne, BP 64182, 31077 Toulouse, France; 2 Department of Biomedical Sciences, Mohammed VI Faculty of Medicine, Mohammed VI University of Sciences and Health, Casablanca, Morocco; 3 Laboratory of Oncopathology, Biology and Environment of Cancer, Mohammed VI Center for Research and Innovation, Rabat, Morocco

**Keywords:** *IgH* locus, p53, Class switch recombination, Germline transcription, *IgH* enhancers, Cancer, Cell biology, Immunology, Molecular biology

## Abstract

Immunoglobulin class switch recombination (CSR) plays an important role in humoral immune response enabling B cells to replace the initial IgM by another antibody class (IgG, IgE or IgA), thus changing the effector functions of antibodies. CSR occurs between highly repetitive switch sequences located upstream of constant gene exons, and is initiated by the Activation-Induced cytidine Deaminase *via* transcription-dependent deamination of single-stranded DNA targets at switch regions. CSR is preceded by germline transcription and is controlled by the super-enhancer 3′ regulatory region (3′RR) in an activation-specific manner. The 3’RR is composed of four enhancers (hs3a, hs1-2, hs3b, and hs4) which act in synergy, and its long-range activity correlates with the enhancers’ transcription. In addition to its function as a tumor suppressor, the p53 transcription factor controls various developmental and cellular processes. This multifaceted function is largely due to its context-dependent transcriptional activity, involving both activation and repression of a myriad of target genes. Despite its potential importance for CSR, which implies multiple layers of transcriptional, epigenetic and DNA break/repair regulations, the role of p53 in CSR is still unclear. Here, by using a mouse line devoid of p53, we show that p53 regulates CSR in an isotype-specific manner. Moreover, we provide evidence that p53 has a dual role in the control of germline transcription as it acts both as an activator and a repressor. Finally, we show that p53 is required for hs4 transcription, suggesting a role for p53 in the regulation of the 3’RR’s transcriptional activity.

## Introduction

Developing B lymphocytes remodel the variable regions of their immunoglobulin (Ig) loci through V(D)J recombination, generating a vast array of antigenic specificities^1–3^. Upon antigen encounter, mature B lymphocytes undergo class switch recombination (CSR) which targets the constant (*C*_*H*_) genes of the Ig heavy chain (*IgH*) locus, ultimately leading to a change of the constant domain of Ig molecules. CSR thus enables activated B cells to switch from the expression of the initial IgM to the expression of downstream isotypes (IgG, IgE or IgA) with novel effector functions^4–7^. CSR relies on various signals received by the B cell (B-cell receptor, cytokines, mitogens, inter-cellular interactions…) and is mediated by particular sequences called switch sequences (or regions) located upstream of the constant exons^4–7^.

Transcription of switch regions (also dubbed germline transcription), which originates from switch promoters (called I promoters), is a pre-requisite for CSR^4–9^. Germline transcription is controlled by various long-range regulatory elements, including enhancers and insulators^8,9^. The major control element is a super-enhancer called 3’ Regulatory Region (3’RR), composed of four enhancers (hs3a, hs1-2, hs3b, and hs4) that act in synergy to activate upstream I promoters in a stimulus-dependent manner^9,10^. Loss of the 3’RR severely impairs CSR by down-regulating germline transcription^11^. The 3’RR enhancer activity correlates with its transcription into enhancer RNAs^8,9^.

Regulation of I promoters involves dynamic conformational changes that are controlled in a developmental stage-, and activation-specific manner^9^. Thus, in resting B cells, the 3’RR engages in stable interactions with Eµ enhancer, located upstream of *C*_*H*_ genes^12^, forming a CSR centre (CSRC)^13^. Upon activation, the primed I promoter is brought to the CSRC where synapsis between Sµ and the partner switch sequence is promoted by Cohesin-mediated loop extrusion^8,13^.

Germline transcription produces long non-coding RNAs that generate secondary structures such as R loops^14^ and G quadruplexes^15^ that provide the substrate^7,16^ for AID (Activation-Induced cytidine Deaminase)^17,18^. AID initiates CSR *via* transcription-dependent deamination of cytosines within single-stranded DNA to uracils^7,16^. Processing of uracils by base excision and mismatch repair pathways leads to double-strand break (DSB) intermediates, at the crucial Sµ donor region and one of the acceptor S regions (Sγ, Sε, Sα). The DSBs are monitored by components of DNA damage response pathway (such as ATM, 53BP1 and H2AX) and repaired by the classical and alternative non-homologous end joining pathways^19,20^, ultimately fusing Sµ and the acceptor S region with the concomitant deletion of the intervening sequence and expression of the newly switched *C*_*H*_ gene^4,6,9^.

In addition to its role as a tumor suppressor, the p53 transcription factor controls multiple developmental and cellular processes in homeostasis^21–23^, and orchestrates the mode of cellular response to various stress factors such as oncogenic activation, telomere erosion and DNA damage. The stress factors influence p53 activity at various levels including its abundance, interaction with co-factors, post-translational modifications and localization^21–23^. Although the precise mechanisms are still debated, it is acknowledged that the multifaceted function of p53 relies, to a large extent, on its context-dependent transcriptional activity, which involves both activation and repression of a broad range of target genes and enhancers, leading to a myriad of cellular responses^21–26^.

In general, p53 activates transcription of adjacent genes by binding to p53-response elements (p53-REs)-containing promoters^23,25,26^, and of distant genes by binding to p53-REs-containing enhancers^23,25–28^. In contrast, the mechanisms underlying p53-mediated repression are less known, and may mostly be indirect: the short-range repression involves factors such as p21, E2F family, Retinoblastoma and micro-RNAs downstream of p53 activation^29^, while long-range repression involves, at least in part, long intergenic non-coding RNAs^30^. More recently, a role for p53 in remodeling chromatin architecture and DNA loops between p53 target enhancers and promoters has been uncovered^31^.

CSR is a process of choice to study the multifaceted function of p53 since CSR involves signal-dependent, local- and long-range transcriptional, epigenetic and 3D chromatin rearrangement processes within the *IgH* constant locus^8^, and a high density of DNA DSBs that recruit DNA damage response and DNA repair pathways^19,20^. Thus, CSR implies multiple layers of regulation, each potentially involving p53. Strikingly, very few studies tackled the role of p53 in CSR. Three groups assayed for CSR to IgG1 as a control in the context of chromosomal translocations associated with CSR^32,33^ or of protection against genomic instability^34^. A single group addressed the role of p53 in CSR to all isotypes^35^.

In order to contribute to a better understanding of the precise function of p53 in CSR, we analyzed the process in a mouse line devoid of p53. We here show that p53 regulates CSR in an isotype-specific manner. Moreover, we provide evidence that p53 has a dual role in the control of germline transcription, acting both as an activator and a repressor of specific *C*_*H*_ genes’ expression, and that p53 regulates the transcriptional activity of the 3’RR in an enhancer-restricted manner.

## Results

### Isotype-specific defect of CSR in the absence of p53

To investigate the effect of p53 deficiency on CSR, negatively sorted, CD43^−^ splenic B cells were stimulated with various cocktails that induce CSR to the different isotypes: LPS (which induces CSR to IgG3 and IgG2b), LPS+IL4 (to IgG1 and IgE), LPS+IFNγ (to IgG2a) and LPS+TGFβ (to IgG2b and IgA), and surface Ig expression was monitored by FACS at days 3 and 4.5 post-stimulation. Surface IgE expression was not assayed because non-specific staining is caused by soluble IgE binding to FcεRII expressed by activated B cells. AID-deficient B cells were included as negative controls as they are unable to initiate CSR.

**Fig. 1:**
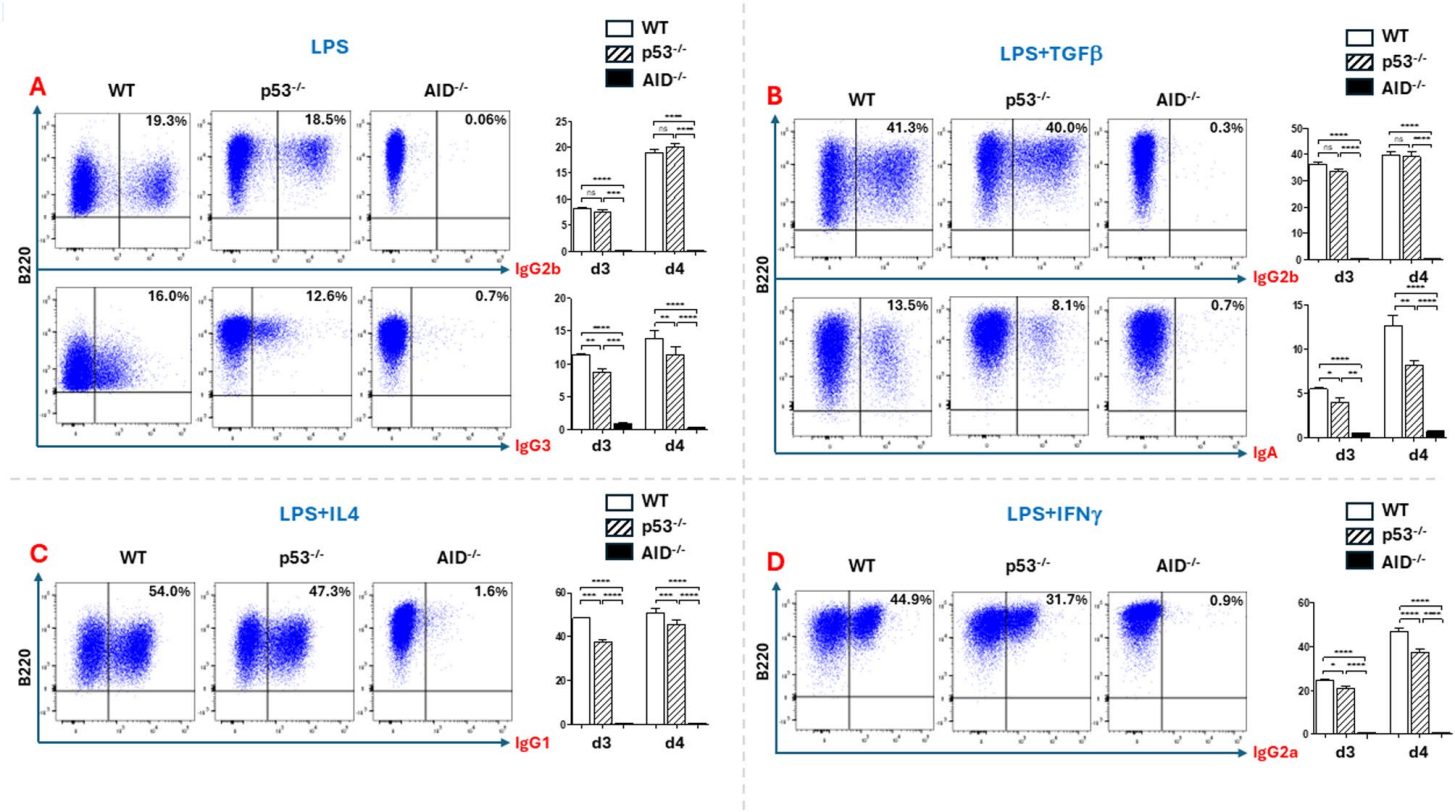
FACS analysis of surface Ig expression. CD43^−^ sorted splenic B cells with the indicated genotypes were induced to switch to IgG3 and IgG2b (LPS stimulation) **(A)**, to IgG2b and IgA (LPS+TGFβ stimulation) **(B)**, to IgG1 (LPS+IL4 stimulation) **(C)**, or to IgG2a (LPS + IFNγ stimulation) **(D)**. At days 3 and 4.5 post-stimulation, the cells were stained with the indicated antibodies. Representative panels at day 4.5 are shown for each stimulation. The statistical data are shown in the right panels. LPS: *n* = 12 for IgG2b, *n* = 9 for IgG3; LPS+IL4: *n* = 14 for IgG1; LPS + IFNγ: *n* = 16 for IgG2a; LPS+TGFβ: *n* = 14 for IgG2b, *n* = 11 for IgA. **** *p* < 0.0001; *** *p* < 0.001; ** *p* < 0.01; ns, not significant.

Upon LPS stimulation, we found that surface IgG2b expression was not altered in p53-deficient B cells (Fig. 1A). In contrast, a moderate but statistically significant decrease of surface IgG3 expression was seen (~ 74% at day 3 and ~ 81% at day 4.5 of WT levels) (Fig. 1A). Somewhat similarly, the levels of surface IgG2b were comparable between p53-deficient B cells and WT controls following LPS+TGFβ stimulation (Fig. 1B), whereas surface expression of IgA was reduced (~ 69% at day 3 and ~ 62% at day 4.5 of WT levels) (Fig. 1B). A slight decrease of surface expression of IgG1 (~ 78% at day 3 and ~ 90% at day 4.5 of WT levels) (Fig. 1C) and a modest reduction of surface IgG2a (~ 85% at day 3 and ~ 79% at day 4.5 of WT levels) (Fig. 1D) was seen on p53-deficient B cells upon LPS+IL4 and LPS+IFNγ stimulations respectively.

Thus, p53-deficiency has no apparent effect on surface expression of IgG2b. In contrast, it leads to a reduction of surface expression of IgG3, IgG1, IgG2a and IgA. Overall, the effect of p53-deficiency is relatively modest with surface expression of IgA being the most severely affected.

### p53-deficiency impairs CSR at the DNA level

FACS analyses revealed that CSR to most isotypes was reduced. However, a defect at the level of Ig surface expression does not necessarily imply an impairment at the switch recombination step. Indeed, the possibility that p53 is involved in post-switch events cannot be formally excluded.

**Fig. 2:**
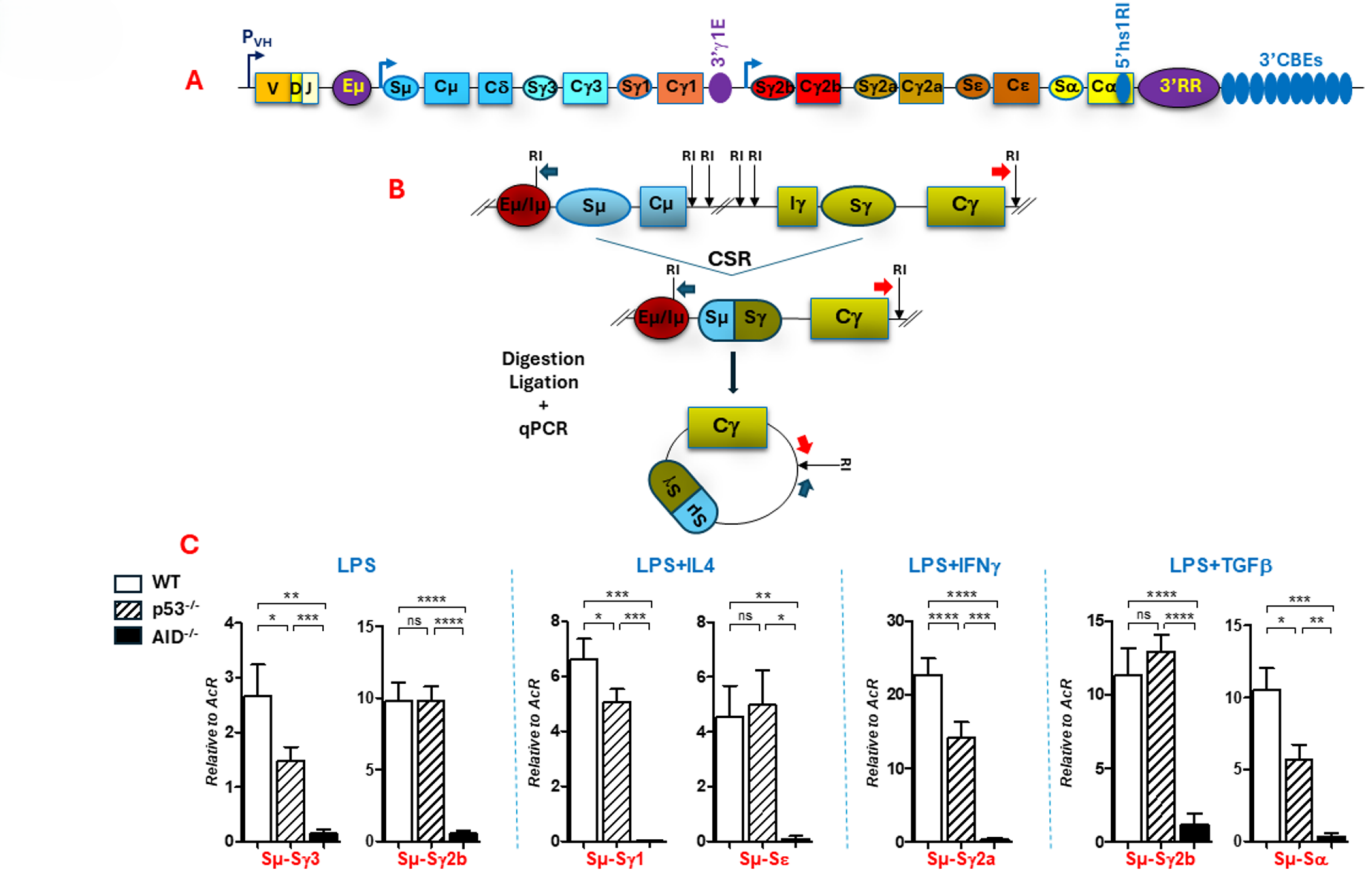
Analysis of CSR at the DNA level by DC-qPCR. **(A)** Scheme of a rearranged murine *IgH* locus. The black arrow indicates transcription from the promoter of the rearranged V(D)J gene. The known regulatory elements of the locus: Eµ and 3’γ1E enhancers, 5’hs1RI insulator, the 3’RR, and the 10 downstream CTCF-binding elements (CBEs) are shown. The blue arrows indicate germline transcription initiation from Eµ/Iµ enhancer/promoter and (as an example) from Iγ2b promoter. **(B)** Outline of the DC-qPCR technique (RI: *Eco*RI). The relative position of the primers is indicated. **(C)** Genomic DNAs were purified from activated splenic B cells (day 4.5) and assayed by DC-qPCR. Quantification of CSR events was performed by qPCR. The *Acetylcholine receptor* gene (*AcR*) which does not undergo rearrangement is used for normalization. LPS: *n* = 8 for Sµ-Sγ3, *n* = 10 for Sµ-Sγ2b; LPS+IL4: *n* = 6 for Sµ-Sγ1, *n* = 7 for Sµ-Sε; LPS + IFNγ: *n* = 10 for Sµ-Sγ2a; LPS+TGFβ: *n* = 8 for Sµ-Sγ2b, *n* = 6 for Sµ-Sα. ****, *p* < 0.0001; ***, *p* < 0.001; **, *p* < 0.01; *, *p* < 0.05; ns, not significant.

To investigate if the reduced surface expression correlates with CSR at the DNA level, we quantified the recombination events between the switch donor Sµ and all the downstream switch acceptors under appropriate stimulations. At day 4.5 post-stimulation, genomic DNAs were purified from activated WT, p53^−/−^, and AID^−/−^ B cells and subjected to DC-qPCR^36^(Fig. 2A, B; Table 1), the AID^−/−^ samples providing the background for the qPCR.

**Table 1:** Primers used in this study.

Primer	Sequence	Tm °C
**Transcription analysis**		
*Germline transcription*		
ImF	CTCTGGCCCTGCTTATTGTTG	61
CmR	GAAGACATTTGGGAAGGACTGACT	61
Ig3F	CAGCCTCAAGGAGATGATGGG	61
Cg3R	CAAGGGATAGACAGATGGGGC	61
Ig1F	GCACACCCCACAGACAAACC	61
Cg1R	ATGGAGTTAGTTTGGGCAGCAG	61
Ig2bF	AAGAGTCCAGAGTTCTCACACACAG	61
Cg2bR	CCAGTTGTATCTCCACACCCAG	61
Ig2aF	CTATGGAACTCTGGGGACCTGG	61
Cg2aR	GTCAAGGTCACTGGCTCAGGG	61
IeF	TAGAGATTCACAACGCCTGGG	61
CeR	CAGGGCTTCAAGGGGTAGAG	61
IaF	GGGTGACTCAGGCTGTTGTGG	61
CaR	AGTGGGTAGATGGTGGGATTTC	61
*3’RR eRNAs*		
HS1-2 F	GGGTGGCTCAACACCCCAGG	61
HS1-2R	GGCTGAGGCAGGCCAAGA	61
HS4F	CAGGCAAGGTGATGTGGATGAGAG	61
HS4R	AGGTCTACACAGGGGCTCTG	61
*Aicda* *transcripts*		
AID-Fw	GGAGACCGATATGGACAGCC	61
AID-Rev	AGAGGTAGGTCTCATGCCGT	61
*p53* *transcripts*		
p53F1	CCTGCCCCAGCCACTCC	61
p53R1	GGCGCGGACACGGCTC	61
*Normalization*		
Act-F1	CAACCGTGAAAAGATGACCCAGAT	61
Act-R1	CAGGATGGCGTGAGGGAGAG	61
**DC-qPCR**		
*Sµ-Sg CSR*		
EcoRI_SmuR	GACCAATAATCAGAGGGAAGAATAATAG	60
EcoRI_Sg3-F	AGCCATCACAATAACCATCTTCCTG	60
EcoRI_Sg1_up1-F	TGAGTAGAAGCAGGGGAGC	60
EcoRI_Sg2b_up1-F	GAAAAAGGGATGGGAAAGCACTC	60
EcoRI_Sg2a_up3-F	GGAAAGGGATGGGAAAGGAC	60
EcoRI_Se-F	GGACACTCAGAATCAACCCTAAG	60
CµHindµR1	GGCTCTCAACCTTGTTCCCTTA	60
CaHindF2	CAGGGAGGGAGAAATACCACC	60
*Normalization*		
EcoRI_AcR-F	GCACACAAACCACTAAACTACTCACTAT	60
EcoRI_AcR-R	TGGTGATAGAGGCAGGAAGA	60
AcR-Fw2H	CGCTGGCAACCCCCTGG	60
AcR-Rev2H	CAGGAAAGAAGAAAGGGGAAATG	60

We found that the frequency of Sµ/Sγ2b recombination events was comparable in LPS-and LPS+TGFβ-activated WT and p53^−/−^ B cells (Fig. 2C). The same holds true for Sµ/Sε events in LPS+IL4 stimulation (Fig. 2C). In contrast, the frequency of Sµ/Sγ3, Sµ/Sγ1, Sµ/Sγ2a and Sµ/Sα recombination events was significantly reduced in p53-deficient B cells under appropriate stimulations (Fig. 2C).

Thus, at the DNA level, p53-deficiency does not affect recombination of Sµ to Sγ2b and Sε. In contrast, it impairs recombination to Sγ3, Sγ1, Sγ2a and Sα.

### Quantification of post-switch transcript levels

**Fig. 3:**
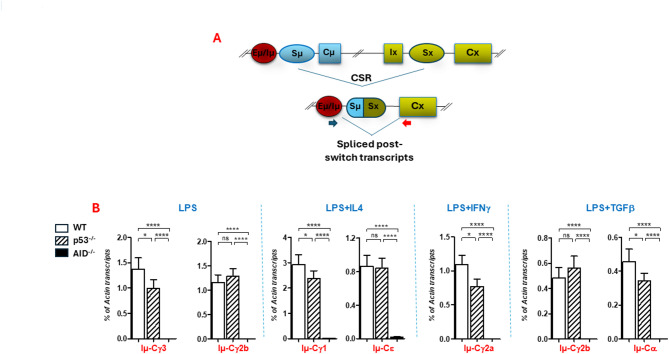
Quantification of post-switch transcript levels. **(A)** The scheme illustrates a recombination event between Sµ and a downstream Sx sequence bringing the associated Cx into proximity of Eµ enhancer, which also acts as a promoter (Iµ) of post-switch transcription. Splicing of Iµ exon onto Cx exons generates mature transcripts (Iµ-Cx) that can be readily quantified. The relative position of the primers used to detect spliced post-switch transcripts is indicated. **(B)** Quantification of post-switch transcript levels in activated splenic B cells with the indicated genotypes and stimulations. At day 4.5 post-stimulation, total RNAs were purified, reverse transcribed and the levels of the indicated post-switch transcripts were quantified by qPCR. AID-deficient B cells are unable to switch (i.e. do not produce post-switch transcripts) and are used as controls for the background level of the qPCR. *Actin* transcripts were used for normalization. LPS: *n* = 15 for Iµ-Cγ3, *n* = 14 for Iµ-Cγ2b; LPS+IL4: *n* = 18 for Iµ-Cγ1, *n* = 15 for Iµ-Cε; LPS + IFNγ: *n* = 12 for Iµ-Cγ2a; LPS+TGFβ: *n* = 16 for Iµ-Cγ2b, *n* = 18 for Iµ-Cα. ****, *p* < 0.0001; *, *p* < 0.05; ns, not significant.

Following recombination of Sµ with a downstream Sx partner, the newly generated Sµ/Sx hybrid sequence falls under the control of Eµ/Iµ enhancer/promoter. In this new, post-germline transcription unit, transcription originates from Eµ/Iµ, extends across Iµ exon, the hybrid Sµ/Sx region, and terminates downstream of the involved *C*_*H*_ gene^37^(Fig. 3A). The levels of post-switch transcripts are thus a good marker of the efficiency of CSR^37^.

In order to quantify post-switch transcript levels, negatively sorted WT, p53^−/−^ and AID^−/−^ B cells were induced to switch for 4.5 days. Total RNA was then purified, reverse transcribed and subjected to qPCR using a forward primer specific of Iµ exon and a reverse primer that pairs specifically with each downstream *C*_*H*_ gene (Fig. 3A; Table 1).

We found that, following appropriate stimulation, the levels of the spliced forms of Iµ-Cγ2b, and Iµ-Cε post-switch transcripts did not vary between activated p53^−/−^ B cells and their WT counterparts (Fig. 3B). In contrast, Iµ-Cγ3, Iµ-Cγ1, Iµ-Cγ2a and Iµ-Cα transcript levels were clearly reduced in activated p53^−/−^ B cells (Fig. 3B). As expected, all post-switch transcripts in AID^−/−^ negative controls were at the background level (Fig. 3B).

Thus, quantification of post-switch transcript levels reveals a reduced recombination of Sµ to Sγ3, Sγ1, Sγ2a and Sα specifically.

Overall, our analyses of CSR in activated p53-deficient B cells, conducted at the level of surface Ig expression, post-switch transcription and DNA recombination, constantly and convergently revealed a reduced efficiency of CSR to IgG3, IgG1, IgG2a and IgA specifically.

### Normal B cell proliferation in the absence of p53

The restricted pattern of CSR impairment in the absence of p53, the large spectrum of p53 effects in homeostasis and the broad range of p53 targets, including genes whose products are involved in cell cycle regulation, led us to hypothesize that p53 deficiency may affect parameters known to be crucial for efficient CSR, so that p53 impact may actually be indirect. One such parameters is B cell proliferation.

Although the mechanisms at play are still unclear, B cell proliferation is required for efficient CSR^38,39^. Therefore, it was important to check whether the isotype-specific reduction of CSR seen in the absence of p53 could be explained, at least in part, by an altered B cell proliferation.

While one might anticipate that this cannot be the case in the context of LPS and LPS+TGFβ stimulations since the levels of IgG2b CSR were normal, and by extrapolation to LPS+IL4 stimulation under which IgE levels did not vary, there still remains the case of LPS+IFNγ stimulation which was reported to enhance IgG2a CSR^35^. To clarify this point, and in order to get a global picture of the effect of p53, we assayed B cell proliferation in the four conditions that cover CSR to all isotypes by FACS, in the presence of CellTrace Violet.

**Fig. 4:**
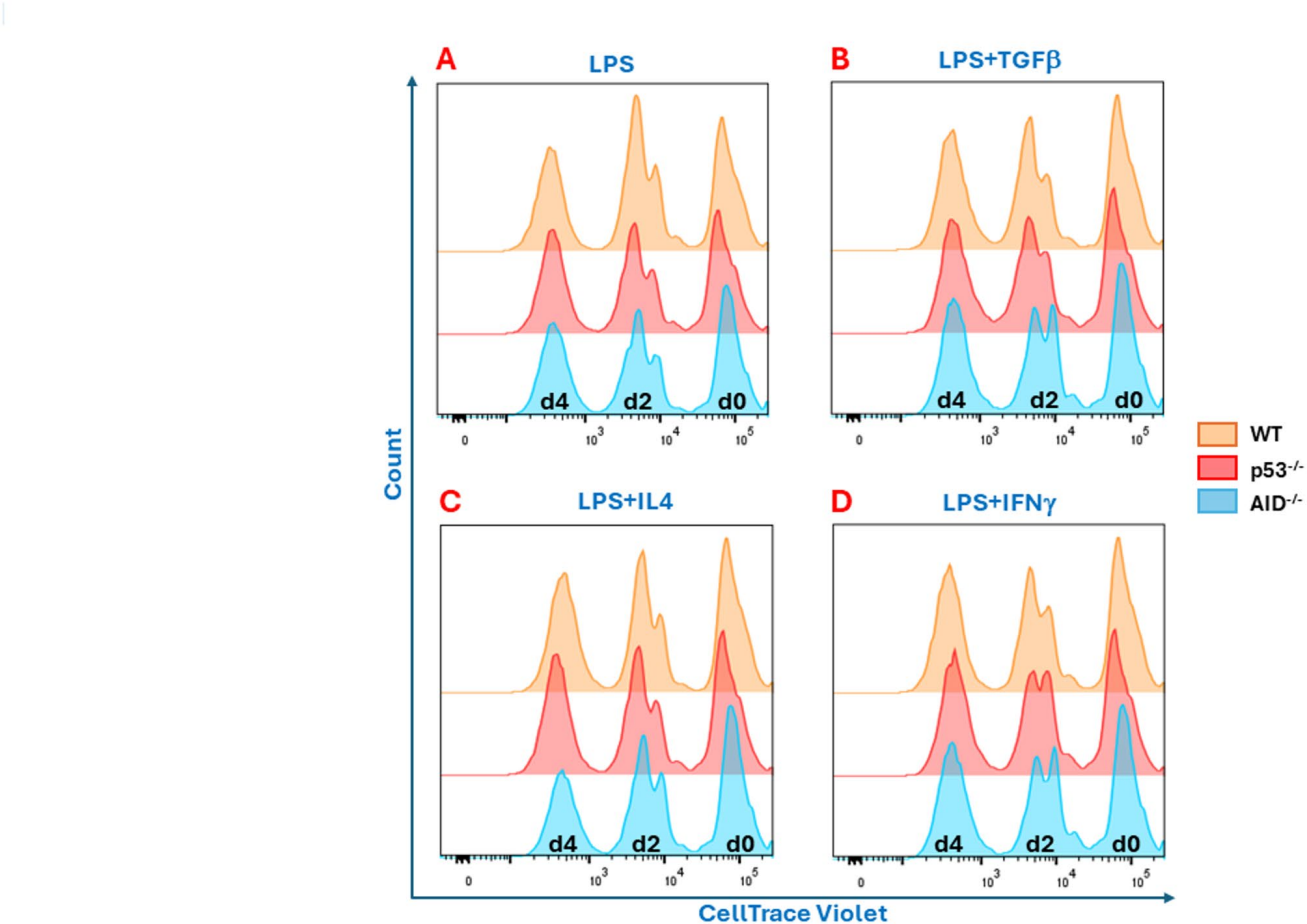
Proliferation assay. **(A-D)** WT, p53^−/−^ and AID^−/−^ splenic B cells were negatively sorted and activated with the indicated stimulations in the presence of CellTrace Violet. FACS analyses were performed at day 0 (prior to stimulation), and at days 2 and 4 post-stimulation. Representative panels are shown for each stimulation (*n* = 3).

We found that in all stimulation conditions tested, proliferation was comparable between WT, p53^−/−^ and AID^−/−^ B cells (Fig. 4A-D).

We conclude that p53 deficiency does not affect B cell proliferation. Consequently, the isotype-specific impairment of CSR in the absence of p53 cannot be ascribed to a defect in B cell proliferation.

### *Aicda* gene expression is not affected by p53 deficiency

AID, encoded by *Aicda* gene, is the key enzyme in CSR as AID-deficient B cells fail to initiate CSR^17,18^. In the context of CSR induction, p53 can potentially regulate *Aicda* gene expression in different ways. For instance, p53 may (directly or indirectly) activate or repress *Aicda* gene regardless of the stimulation as it may activate or repress *Aicda* gene in a stimulus-dependent manner. Hence, p53-deficiency can potentially alter CSR efficiency indirectly by impacting *Aicda* gene expression.

Therefore, we quantified *Aicda* transcript levels in p53-deficient B cells activated with LPS, LPS+IL4, LPS+IFNγ, and LPS+TGFβ. We did not extend our analyses to AID as it has been shown that AID production correlates well with *Aicda* gene expression^35,40,41^.

**Fig. 5:**
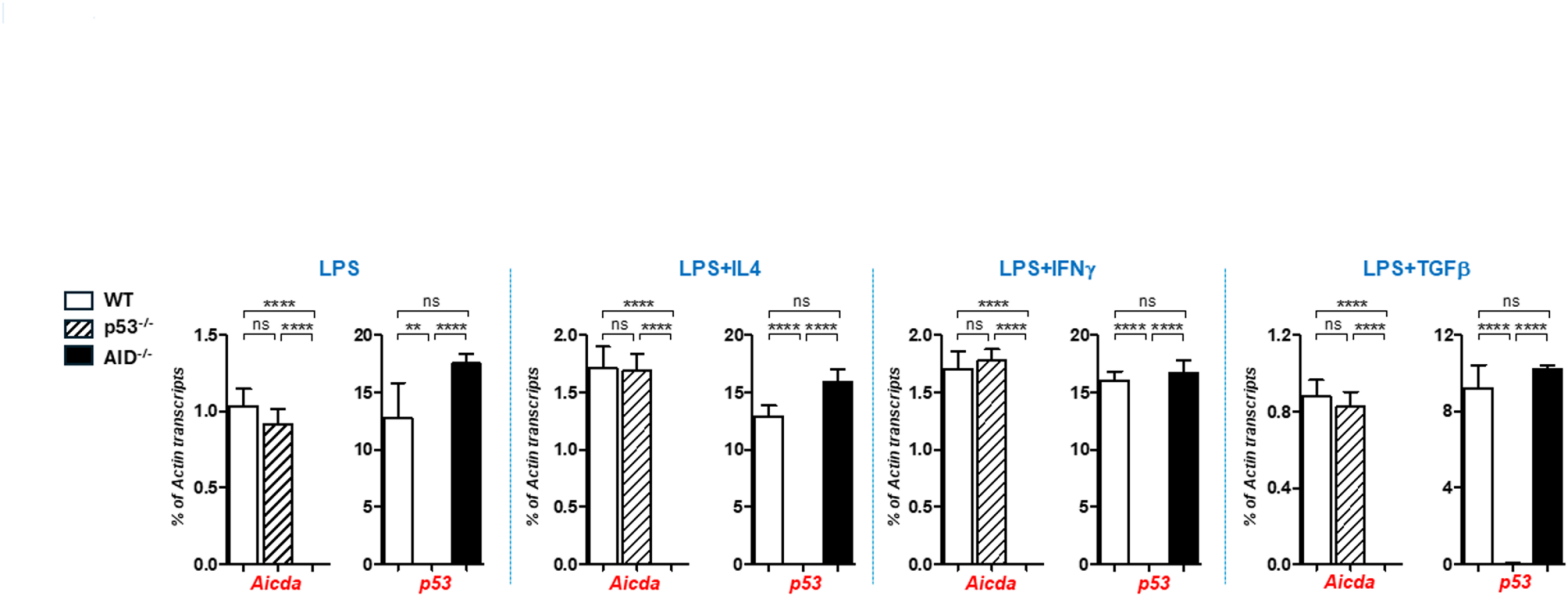
Quantification of *Aicda* and *p53* transcript levels. The spliced forms of *Aicda* and *p53* transcripts were quantified in activated splenic B cells with the indicated genotypes and stimulations. At day 2 post-stimulation, total RNAs were purified, reverse transcribed and the indicated transcript levels were quantified by RT-qPCR. LPS: *n* = 12 for *Aicda*, *n* = 5 for *p53*; LPS+IL4: *n* = 14 for *Aicda*, *n* = 5 for *p53*; LPS + IFNγ: *n* = 14 for *Aicda*, *n* = 5 for *p53*; LPS+TGFβ: *n* = 14 for *Aicda*, *n* = 5 for *p53*. ****, *p* < 0.0001; **, *p* < 0.01; ns, not significant.

At day 2 post-stimulation, total RNA was purified, reverse-transcribed and subjected to qPCR using primer pairs specific of the spliced forms of *Aicda* and *p53* transcripts (Table 1). As expected, *Aicda* and *p53* transcripts were undetectable in AID- and p53-deficient B cells respectively. *p53* transcript levels did not vary between WT and AID-deficient B cells in all stimulation conditions (Fig. 5). More importantly, *Aicda* transcript levels were comparable between activated WT and p53-deficient B cells regardless of the stimulation condition (Fig. 5).

Thus, in the context of CSR induction, p53 does not play an obvious role in the control of *Aicda* gene expression. Consequently, the decreased levels of CSR to most isotypes cannot be explained by reduced levels of AID.

### Dual role of p53 in the regulation of germline transcription

It is well established that germline transcription is absolutely required for CSR, and mutations that abolish or reduce germline transcription of a given *C*_*H*_ gene generally abrogate or reduce CSR to that particular gene. Nonetheless, exceptions to this “rule” have been reported, in particular for IgA CSR^8^. The reduced levels of CSR to IgG3, IgG1, IgG2a and IgA thus led us to investigate how germline transcript levels correlate with CSR efficiency in the absence of p53.

**Fig. 6:**
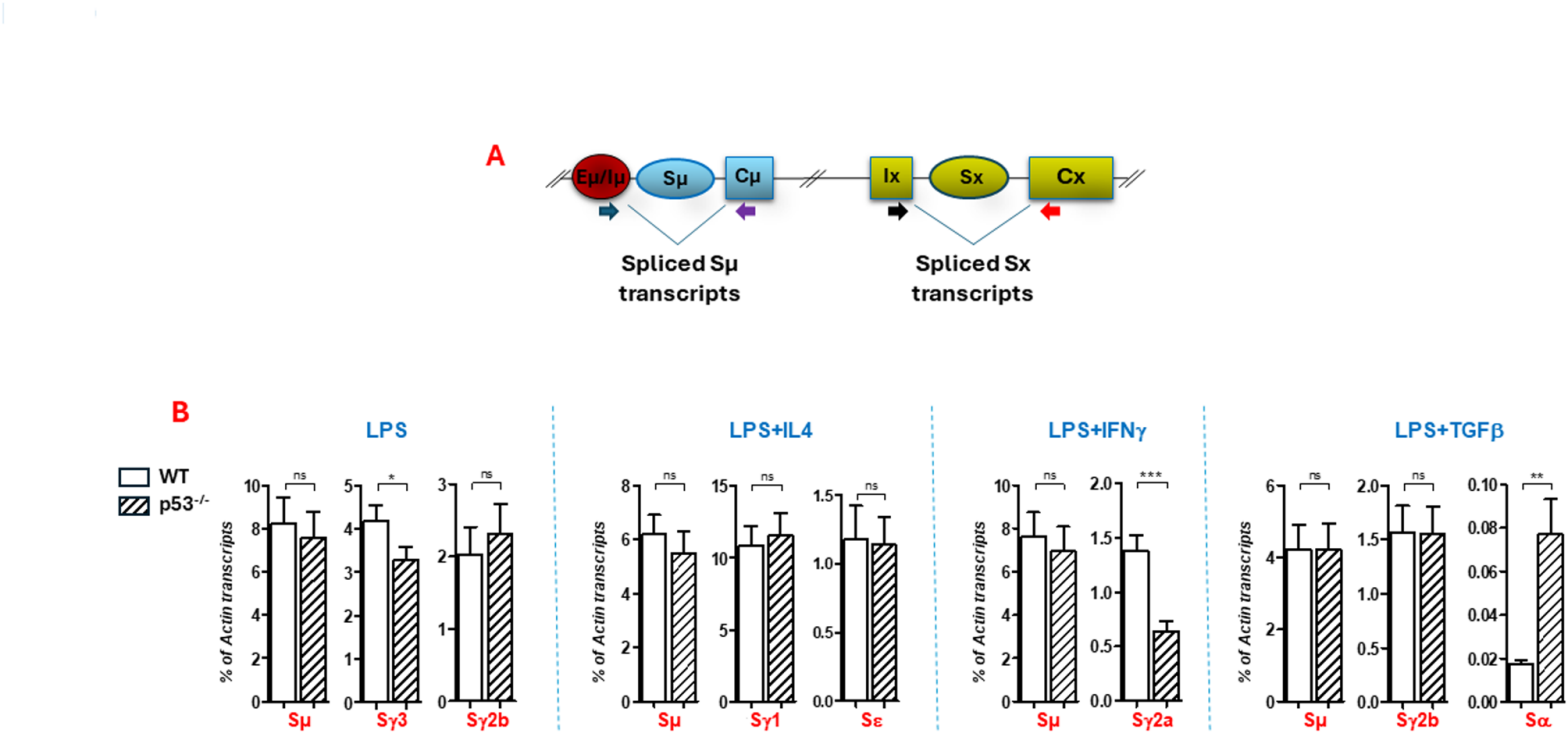
Quantification of germline transcript levels. **(A)** The scheme represents *Cµ* gene and a downstream constant gene, x stands for any isotype. The relative position of the primers used to detect spliced germline transcripts is indicated. **(B)** Quantification of germline transcript levels in activated splenic B cells with the indicated genotypes and stimulations. At day 2 post-stimulation, total RNAs were purified, reverse transcribed and the levels of the indicated germline transcripts were quantified by qPCR. LPS: *n* = 12 for Sµ, *n* = 15 for Sγ3, *n* = 15 for Sγ2b; LPS+IL4: *n* = 12 for Sµ, *n* = 15 for Sγ1, *n* = 15 for Sε; LPS + IFNγ: *n* = 12 for Sµ, *n* = 13 for Sγ2a; LPS+TGFβ: *n* = 12 for Sµ, *n* = 15 for Sγ2b, *n* = 15 for Sα. ***, *p* < 0.001; **, *p* < 0.01; *, *p* < 0.05; ns, not significant.

To this end, negatively sorted CD43^−^ splenic B cells were stimulated with various cocktails that induce the different I promoters: LPS stimulation which induces Iγ3 and Iγ2b, LPS+IL4 stimulation which induces Iγ1 and Iε, LPS+IFNγ stimulation which induces Iγ2a, and LPS+TGFβ stimulation which induces Iγ2b and Iα. At day 2 post-stimulation, the spliced forms of germline transcript levels were quantified by RT-qPCR (Fig. 6A).

It will be seen from Fig. 6B that Sµ transcript levels did not vary between activated p53^−/−^ and WT controls regardless of the stimulation cocktail. Thus, a potential defect in CSR cannot be due to reduced levels of transcription across the switch donor region. Likewise, the levels of Sγ1, Sγ2b, Sε transcripts were comparable between p53^−/−^ and WT controls. In contrast, Sγ3 and Sγ2a transcript levels were variously reduced in activated p53^−/−^ B cells (Fig. 6B). Unexpectedly, Sα transcript levels were clearly increased in activated p53^−/−^ B cells (Fig. 6B).

Thus, p53 is dispensable for transcription of Sγ1, Sγ2b and Sε. Importantly, while p53 acts as a transcriptional activator of Sγ3 and Sγ2a transcription, it acts as a repressor of Sα transcription. We conclude that p53 has a dual role in its isotype-specific control of germline transcription (See Fig. 8).

**Fig. 7:**
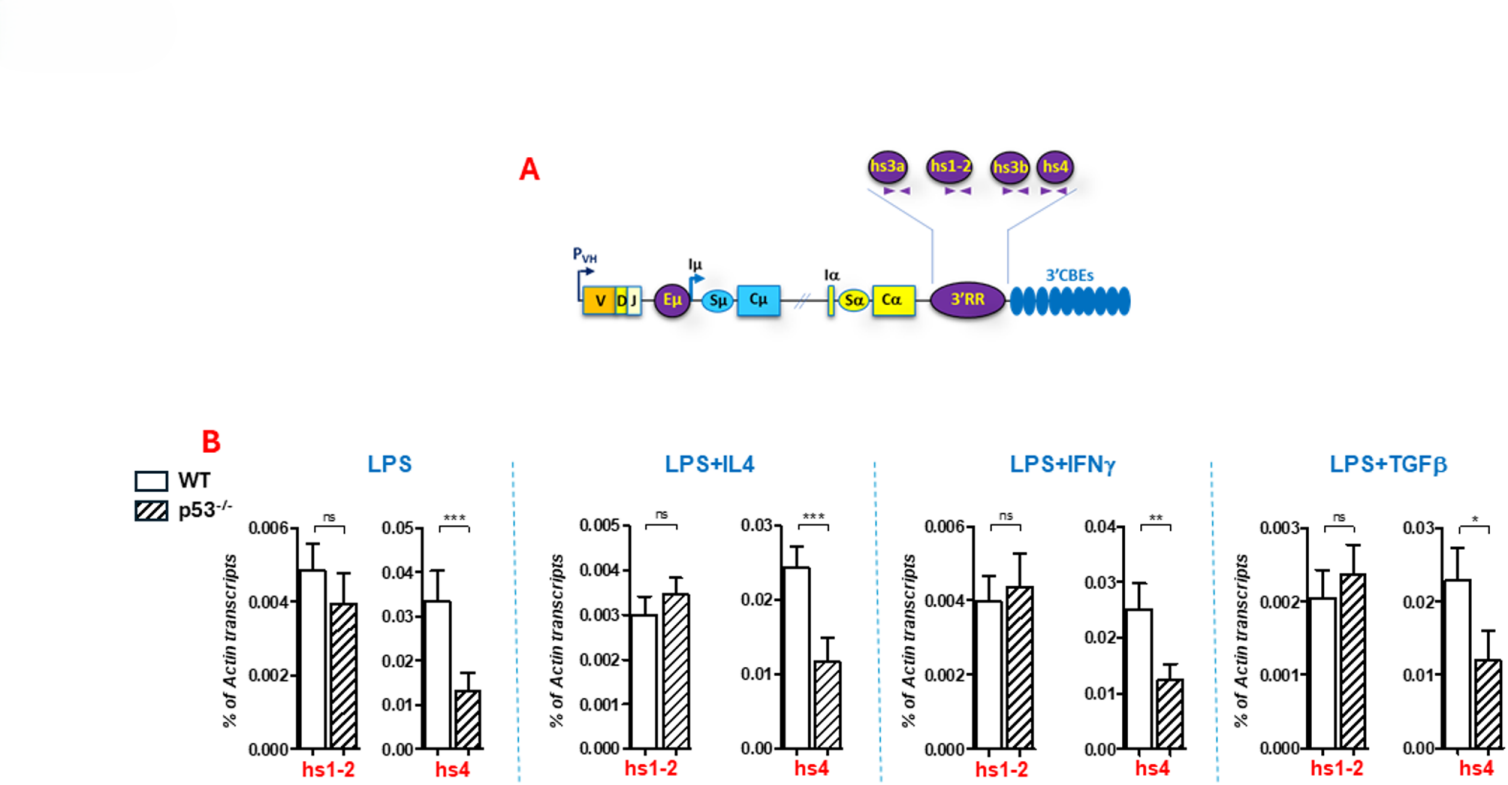
Quantification of 3’RR eRNAs levels. **(A)** The scheme depicts the 3’RR enhancers. The relative position of the primers used to detect 3’RR eRNAs is indicated (see text for additional details). **(B)** Quantification of pre-switch transcript levels in activated splenic B cells with the indicated genotypes and stimulations. At day 2 post-stimulation, total RNAs were purified, reverse transcribed and the levels of the indicated eRNAs were quantified by qPCR. Minus RT controls were included throughout. LPS: *n* = 12 for hs1-2, *n* = 15 for hs4; LPS+IL4: *n* = 14 for hs1-2, *n* = 15 for hs4; LPS + IFNγ: *n* = 15 for hs1-2, *n* = 15 for hs4; LPS+TGFβ: *n* = 15 for hs1-2, *n* = 14 for hs4. ***, *p* < 0.001; **, *p* < 0.01; *, *p* < 0.05; ns, not significant.

**Fig. 8:**
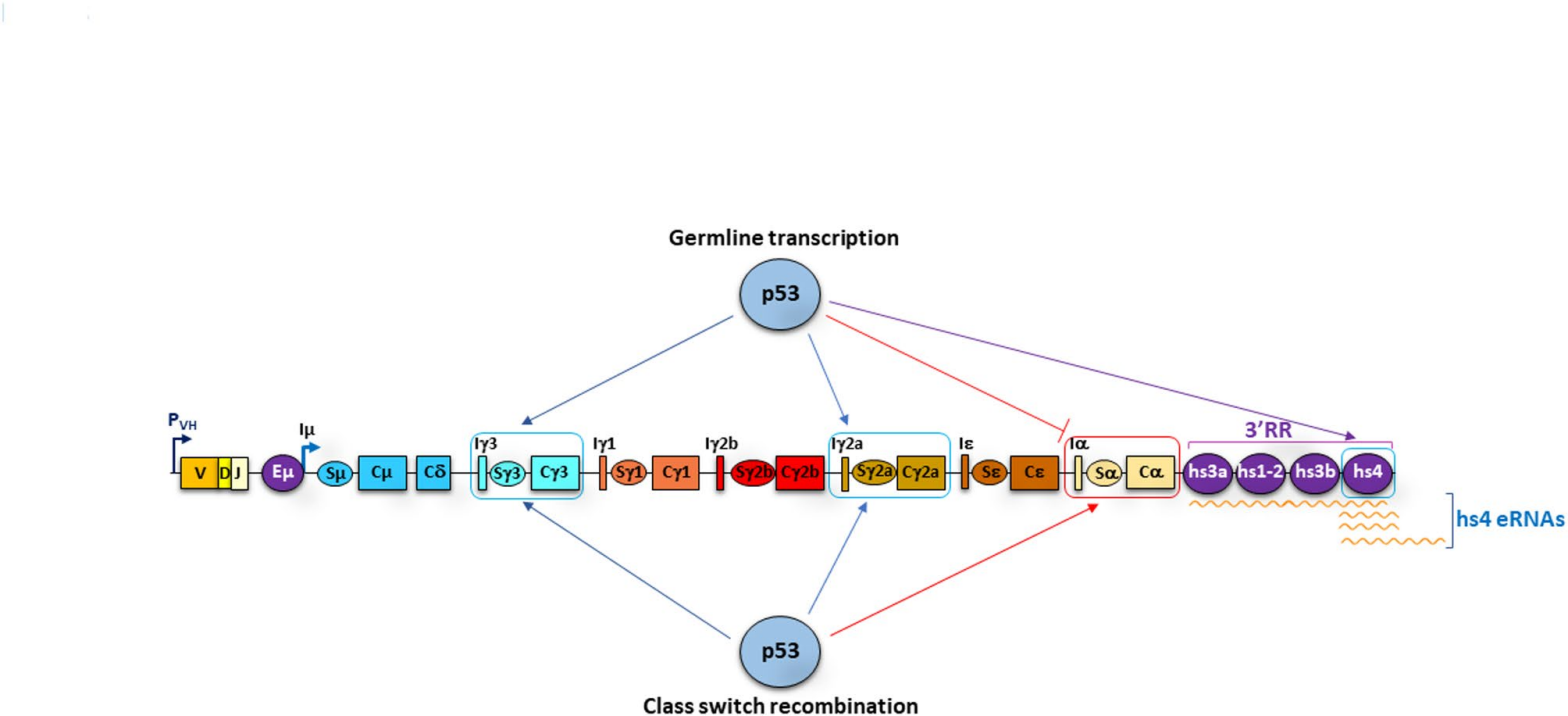
A scheme recapitulating the main effects of p53 at the *IgH* locus. p53 activates Sγ3 and Sγ2a transcription but represses Sα transcription. In the absence of p53, CSR to IgG3, IgG2a and IgA is reduced. CSR to IgG1 (not depicted) is slightly reduced despite normal Sγ1 transcription. p53 is also involved in the transcriptional activity within the 3’RR by specifically targeting hs4: in the absence of p53, hs4 eRNAs levels are reduced (see text for details).

### p53 regulates transcription within the 3’RR in an enhancer-restricted manner

The 3’RR is the major control element of germline transcription^8–10^, and it is increasingly acknowledged that the transcriptional activity within the 3’RR correlates with its long-range control of I promoters^8,9^. The transcriptional activity within the 3’RR produces sense and anti-sense transcripts (i.e. 3’RR eRNAs)^13,42^, but it is still unclear whether the crucial eRNAs for the 3’RR activity originate from the 3’RR enhancers themselves, initiate at adjacent elements but elongate across these enhancers, or both. While some progress has been achieved in the identification of the initiation start sites of specific 3’RR eRNAs^42,43^, the precise maps and the fine details of the 3’RR eRNAs structures are still lacking. Keeping this in mind, and for simplicity, we will assign the eRNAs to specific enhancers based on the origin of the primer pair sequences (Fig. 7A).

Because the deletion of p53 impacted Sγ3, Sγ2a and Sα transcription known to be controlled by the 3’RR^8^, we sought to investigate how this isotype-specific effect correlated with the transcriptional activity within the 3’RR. To this end, hs3a, hs1-2, hs3b and hs4 eRNA levels were quantified by RT-qPCR at day 2 post-stimulation.

We found that the levels of hs3a and hs3b eRNAs (not shown) as well as hs1-2 eRNAs (Fig. 7B) were not affected by p53-deficiency. In contrast, hs4 eRNA levels were reduced in all stimulation conditions (Fig. 7B).

We conclude that p53 is required for optimal transcription of hs4 enhancer specifically (see Fig. 8).

## Discussion

In this study, we showed that p53 deficiency reduces at various levels CSR to IgG3, IgG1, IgG2a and IgA. This isotype-restricted decrease correlates with the corresponding germline transcription in a complex manner. While reduced IgG3 and IgG2a CSR correlates with reduced Sγ3 and Sγ2a transcript levels respectively, decreased IgA CSR occurs despite increased Sα transcript levels, indicating a dual role for p53: an activator of Sγ3 and Sγ2a transcription and a repressor of Sα transcription. On the other hand, the modestly decreased IgG1 CSR is seen despite normal Sγ1 transcription suggesting a role for p53 at a step beyond Sγ1 transcription. Additionally, we showed that p53 is involved in the regulation of hs4 transcription specifically, suggesting a role for p53 in the regulation of the transcriptional activity within the 3’RR. Finally, in agreement with previous reports^33–35^, we found that p53 deficiency does not perturb B cell proliferation or AID levels.

As mentioned previously (see introduction), very few studies addressed the role of p53 in CSR. In the context of CSR-associated chromosomal translocations or genomic stability, three studies reported that IgG1 CSR was apparently unaffected by p53 deficiency^32–34^. More pertinent to our findings, a third study which directly addressed the role of p53 in CSR to all isotypes reported that CSR to IgG2a was dramatically enhanced, and CSR to IgG2b and IgA was slightly increased in the absence of p53^35^. The increased IgG2a CSR was seen despite normal Sγ2a transcription, whereas the levels of Sγ2b and Sα transcripts were not reported^35^. The reasons underlying the discrepancies between these studies, especially that of Guikema et al.^35^, and ours are unclear but may relate to the stimulation protocols and the resulting frequencies of CSR, and/or to the sensitivity of the methods used to quantify germline transcripts^35^.

It is established that p53 transcriptional activity, be it activation or repression of target genes, depends on the cellular context including cell-type, cell state, and the nature of activation signals^22,25,26^. At first glance, our findings extend this feature to the *IgH* locus: under different stimuli (LPS, LPS+INFγ and LPS+TGFβ), p53 activation would lead to different outcomes, though within the same locus: activation of *Cγ3* and *Cγ2a* genes, and repression of *Cα* gene. However, a close inspection of the data reveals a more complex situation. The *IgH* locus is the site of both transcription and recombination. Since in CSR, germline transcription precedes recombination, the simplest explanation is that the reduced levels of IgG3 and IgG2a CSR result from reduced Sγ3 and Sγ2a transcription. However, this does not apply to IgG1 CSR for which Sγ1 transcription is unaffected and to IgA CSR for which Sα transcription is enhanced. This cannot be *solely* due to the type of stimulation, the unaffected IgG2b and IgE CSR argue against it. We suggest that p53 is involved, through as yet unknown mechanism, in Sµ/Sγ1 recombination step though this role appears to be rather marginal. The case of IgA is more complex and suggests that there is likely something specific to Sα transcription and/or Sµ/Sα recombination that distinguishes it from the other isotypes (see below).

One possibility to account for the isotype-specific effect of p53 could be the nature and/or presence of p53-REs. The consensus sequence of p53-REs is degenerate, and is made up of two half-sites separated by a spacer of 0 to 21 base pairs (RRRCWWGYYY-spacer-RRRCWWGYYY, where R=purine, Y=pyrimidine and W = A or T)^22,23^. In this regard, bioinformatic analyses of the *IgH* constant locus sequence using JASPAR database revealed the presence of putative p53-REs at different sites of all downstream *C*_*H*_ genes (though none at the corresponding I promoters), be they affected by p53 deficiency or not. Likewise, p53-REs were found in hs4 enhancer, but also in the unaffected hs1-2 enhancer (data not shown). It has long been noted that the presence of a p53-RE does not imply p53 binding, and p53 binding does not imply transcriptional regulation of the adjacent gene. The situation becomes more complex when one considers the nature of the consensus p53-RE and the observation that any variation on each of its structural features can impact p53 binding and activity^22,23,26^. Last but not least, and due to the wide spectrum of p53 targets, it is presently difficult to ascertain whether p53 acts directly or indirectly on the *IgH* constant locus.

The 3’RR-mediated long-range control of CSR correlates with the transcriptional activation of the 3’RR’s enhancers, but the exact role of the resulting eRNAs in CSR is still unclear^9^. We found that p53 deficiency leads to a marked decrease of hs4 eRNAs levels specifically, regardless of the type of stimulation, suggesting that p53 is required for optimal hs4 transcription, and that it is involved in the regulation of the transcriptional activity within the 3’RR, potentially affecting its long-range activity. Two critical points need to be faced at the outset: (1) Deletion of hs4 enhancer has seemingly no effect on CSR to all isotypes^44^. However, germline transcription was not analyzed in these mice. It is conceivable that the reduced levels of hs4 eRNAs in the absence of p53 have no impact on 3’RR’s activity as a whole, being for instance compensated by the other eRNAs. Nonetheless, there is evidence that individual enhancer-associated eRNAs can impact 3’RR’s activity^42,45^, and at least in the case of the CH12F3-2 A cell line, which switches to IgA exclusively, reducing the levels of hs4 eRNAs specifically caused a decrease in IgA CSR^43,46^. (2) Besides the 3’RR-dependent Sγ3, Sγ2a and Sα transcription, transcription of the other 3’RR-dependent switch regions was normal (this study). This apparent discrepancy could be rationalized by the notion that individual 3’RR enhancers display preferential interactions with I promoters^47,48^. This would imply that p53 is involved in the preferential interaction between hs4 and Iγ3, Iγ2a and Iα promoters. It is plausible that, because of the distance, expression of the remote *Cγ3* and *Cγ2a* genes is more dependent on the long-range activation by the 3’RR involving chromatin loop formation. Therefore, reduced Sγ3 and Sγ2a transcription would result, at least in part, from reduced hs4 eRNAs. For the more proximal *Cα* gene, lying right upstream of the 3’RR, the preferential interaction with hs4 would not be the most critical parameter but rather local factors such as spreading of active histone marks from the 3’RR^49^.

Perhaps the most unexpected finding of this study is the reduced IgA CSR despite enhanced Sα transcription. It is known that TGFβ signaling phosphorylates SMAD2 and SMAD3, which then bind SMAD4 and Runx3 transcription factors to form active complexes that translocate to the nucleus and activate Iα promoter^50–54^. Interestingly, previous studies showed that p53 binds to the complex and can convey either activation or repression^55–59^. It is thus conceivable that p53 binds the SMAD/Runx complex in TGFβ-activated B cells and down-modulates Sα transcription.

One of the yet unsolved issues in the field of CSR relates to the question as to whether germline transcription is all that is needed for Sµ/Sx recombination, regardless of switch sequence identity, or that isotype-specific factors are involved (in addition to germline transcription). Switch sequence replacement experiments at the endogenous *IgH* locus, using closely related Sγ sequences, tended to downplay such isotype-specific requirement^60^, contrasting with other studies, based on extrachromosomal vectors, which concluded to the existence of isotype-specific factors^61–65^. In particular, it was proposed that the ubiquitous histone methyltransferase Suv39h1, which catalyzes the histone modification H3K9me3, is a Sα-specific factor^65^. Strikingly, Suv39h1-deficient B cells displayed reduced CSR to IgA exclusively^65^. Previous studies found H3K9me3 mark on most switch sequences (Sα was not studied)^66,67^. Upon activation, this mark is erased from transcribed downstream switch sequences but is retained at Sµ^67^. Interestingly, *Suv39h1* is a target of p53 repression^68,69^. It is tempting to speculate that in the absence of p53, H3K9me3 mark persists on Sα thus limiting Sµ/Sα recombination. Clearly, additional studies are needed to test these hypotheses.

## Materials and methods

### Mice and ethical guidelines

WT 129Sv1 mice were purchased from Charles River. p53-deficient mice were provided by B. Ryffel (INEM, CNRS UMR7355, Orléans). AID-deficient mice were provided by T. Honjo, through C-A. Reynaud and J-C. Weill. p53- and AID-deficient mice were back-crossed into 129Sv1 background for at least 10 generations before use. Throughout this study, the mice were maintained in specific pathogen-free zone. All the mice were 6–8 weeks-old. Mice (30–35 g) were euthanized by inhalation of carbon dioxide (CO₂) using an automated euthanasia box (Tem Sega), with a 2 min induction phase (air/CO₂) involving a gradual increase in CO₂ concentration up to 70%, followed by an additional 2 min euthanasia phase. After emptying the CO2 (which lasts 3 min), the procedure is followed by cervical dislocation to confirm death. All the experiments with mice were carried out according to the CNRS Ethical guidelines and were approved by the Regional Ethical Committee (Accreditation N° F31555005), and complying with ARRIVE guidelines.

### Antibodies and cytokines

APC-conjugated anti-B220, PE-conjugated anti-IgG1, PE-conjugated anti-IgG2b, PE-conjugated anti-IgG2a antibodies, IL4, IL5, TGFβ1, and BLyS were purchased from Biolegend. Dextran-conjugated anti-IgD was from Fina Biosolutions, PE-conjugated anti-IgG3, PE-conjugated anti-IgA from eBioscience, IFNγ from R&D Systems, and Lipopolysaccharide (LPS) from Sigma.

### Induction of germline transcription and CSR

Single cell suspensions from spleens were obtained by standard techniques and splenic B cells were negatively sorted using CD43-magnetic microbeads and MS columns (Miltenyi). To induce germline transcription and CSR, CD43^−^ splenic B cells were cultured for 2 days (germline transcription), 3 and 4.5 days (CSR), at a density of 5 × 10^5^ cells/ml in the presence of LPS (25 µg/ml) + anti-IgD-dextran (3 ng/ml) (hereafter LPS stimulation), LPS (25 µg/ml) + anti-IgD-dextran (3 ng/ml) + IFNγ (20 ng/ml) (LPS + IFNγ stimulation), LPS (25 µg/ml) + anti-IgD-dextran (3 ng/ml) + IL4 (25 ng/ml) (LPS+IL4 stimulation), or LPS (25 µg/ml) + anti-IgD-dextran (3 ng/ml) + IL4 (10 ng/ml) + IL5 (5 ng/ml) + BLyS (5 ng/ml) + TGFβ (2 ng/ml) (LPS + TGFβ stimulation).

### Cell proliferation assay

The assay was conducted according to the manufacturer’s instructions (Invitrogen). Briefly, Purified CD43^−^ splenic B cells were washed with ice-cold PBS. After centrifugation, cell concentration was adjusted to 1 × 10^6^ cells/ml, and the cells were incubated with freshly diluted CellTrace Violet (final concentration 1µM) at room temperature for 20 min, protected from light. After addition of complete culture medium and incubation at room temperature for 5 min, the cells were pelleted, resuspended in a pre-warmed complete culture medium at a concentration of 1 × 10^6^ cells/ml, and incubated for 10 min at room temperature. The cells were then analyzed by FACS (day 0), or stimulated with the various CSR-inducing cocktails, and assayed for proliferation at day 2 and day 4 post-stimulation.

### Fluorescence-activated cell sorting (FACS) analyses of CSR

At day 4.5 post-stimulation, B cells were washed and stained with anti-B220-APC and either anti-IgG3-PE, anti-IgG1-PE, anti-IgG2b-PE, anti-IgG2a-PE, or anti-IgA-PE. Activated B cells from AID-deficient mice (unable to initiate CSR) were included throughout as negative controls. Data on 1 × 10^4^ viable cells were obtained using a BD LSR Fortessa X-20 flow cytometer.

### Quantification of transcript levels by RT-qPCR

Total RNAs were prepared using a commercial kit (Zymo Research), reverse transcribed (Ozyme), and subjected to qPCR using SsoAdvanced Universal SYBR Green Supermix (BioRad). *Actin* transcripts were used for normalization. For eRNAs’ quantification, minus RT controls were included for all samples.

### DC-qPCR

Genomic DNAs were purified from WT, p53^−/−^ and AID^−/−^ splenic B cells at day 4.5 post-stimulation. After *Eco*RI (for CSR to Sγ and Sε) or *Hind*III (CSR to Sα) digestion and circularization, ligation products were subjected to qPCR. *Acetylcholine receptor* gene was used for normalization. Note that the Sγ3- and Sε-forward primers are located downstream of the *γ3* and *ε* constant genes respectively, and that the Sα-forward primer is located downstream of Cα3 exon. Therefore, all CSR events to Sγ3, Sε and Sα are presumably detected. Sγ1-, Sγ2b- and Sγ2a-specific forward primers are located upstream of the *Eco*RI site towards the end of Sγ1, Sγ2b and Sγ2a regions; thus, the few CSR events potentially involving the short remaining S sequences downstream of the *Eco*RI site are not detected.

### Primers

All the primers used in this study are listed in Table 1.

### Statistical analysis

Results are expressed as mean ± SD (GraphPad Prism) and overall differences between values were evaluated by *t*-test with Mann-Whitney Post-test. The difference between means is significant if *p* value < 0.05 (*), very significant if *p* value < 0.01 (**), extremely significant if *p* value < 0.001 (***) or if *p* value < 0.0001 (****).

## Data Availability

All data generated or analyzed during this study are included in this manuscript.

## Abbreviations

3’RR: 3’ Regulatory region
CSR: Class switch recombination
eRNA: Enhancer RNA
IgH: Immunoglobulin heavy chain
LPS: Lipopolysaccharide
IL: Interleukin
